# Effect of neuromuscular electrical stimulation associated with swallowing-related muscle training for post-stroke dysphagia: A protocol for systematic review and meta-analysis

**DOI:** 10.1097/MD.0000000000025108

**Published:** 2021-03-19

**Authors:** Bosong Du, Yan Li, Bingran Zhang, Wenjun Zhao, Li Zhou

**Affiliations:** Chengde Central Hospital, Chengde, Hebei Province, China.

**Keywords:** dysphagia, meta-analysis, neuromuscular electrical stimulation, protocol, swallowing rehabilitation training, systematic review

## Abstract

**Background:**

Swallowing dysfunction is a common dysfunction after stroke, and its incidence exceeds 50%. Aspiration pneumonia and malnutrition induced by dysphagia not only cause psychological shock to patients after stroke, but also burden the medical payment. Neuromuscular electrical stimulation, which stimulates the cortex and cortical bulb pathways to improve swallowing function, has been one of the emerging treatments for the post-stroke deglutition disorder. These therapy operators require the proficiency in professional knowledge, limiting clinical large sample studies, so there is an absence of evidence-based medicine. The research is to evaluate the effectiveness of neuromuscular electrical stimulations combined with swallowing-related muscle training to treat swallowing dysfunction after stroke.

**Methods:**

Computer retrieval performed in the 9 databases, including PubMed, Embase, Web of science, Cochrane Library, ClinicalTrials, China Biomedical Literature Database (CBM), China Knowledge Network Database (CNKI), Wanfang Database (WanFang), and China VIP Database (VIP). Taking the published literature from the establishment of the database until December 20, 2020. Literature searching is related to neuromuscular electrical stimulation randomized controlled trials on the effect of swallowing in stroke. In addition, we will do the manual search in Baidu Academic and Google Academic database as a supplementary search. The correlative randomized controlled clinical studies retrieval time range from the establishment of the database to December 20, 2020. Two investigators will screen the literature according to the inclusion and exclusion criteria independently, during that period they will evaluate the quality of the included studies and extract data from studies. The extracted data are dichotomous data will be represented by relative risk, continuous data will be represented by mean difference or standard mean deviation. If there exists heterogeneity and the final data summary analysis select random effect model. On the contrary, the fixed effect model is selected. Then, RevMan5.3 software was used when analyzing included literature. Meanwhile, the analysis results were illustrated by drawing.

**Results:**

This review will summarize available trials aimed at providing a comprehensive estimation of effectiveness of neuromuscular electrical stimulation associated with swallowing muscle training for post-stroke dysphagia.

**Conclusion:**

This review based on a comprehensive analysis of currently published randomized controlled trials on post-stroke dysphagia, that provide reliable evidence-based medicine evidence for the efficacy of neuromuscular electrical stimulation associated with swallowing rehabilitation training.

**Registration number:**

INPLASY202110009.

## 1 Introduction

Swallowing transport food and water from mouth to stomach^[1]^ and protects the airway from aspiration.^[2]^ The whole swallowing divided into oral preparatory phase, oral propulsive phase, pharyngeal, and esophageal phase.^[3]^ Dysphagia can occur in any process of swallowing.^[4]^ The swallowing disorder can cause by abnormal structure and function, among which neurologic disease and stroke are the important disease factors of causing swallowing disorder.^[4,5]^ After a stroke, oropharyngeal dysphagia observed during the first 2 to 4 weeks with a more than prevalence of 50%,^[6,7]^ one systematic review and meta-analysis reveals that dysphagia occurrence rate is 36.3% in Asia.^[8]^ Malnutrition, dehydration, and stroke related pneumonia which are all related to deglutition disorder.^[9–11]^ So swallowing disorder can seriously hamper patients’ recovery and this kind of sequela of apoplexy brings a huge psychological shock to patients who can eat normally before stroke.^[12]^ At the same time, a burden of stroke treatment will increase, and mortality in post-stroke patients with dysphagia, dehydration, malnutrition, and asphyxia will significantly mount.^[13]^

In recent years, neuromuscular electrical stimulation (NMES) as a professional technical means of therapy of swallowing dysfunction after stroke has been utilized in clinicals which can improve swallowing function by activating swallowing-related central motor cortex and corticobulbar pathways.^[14,15]^ With various clinical trials proceed, there are differences in research protocols and evaluations of efficacy. That will cause the absence of uniformity in the research results, and the delay of clinical application for this therapy. Therefore, this review will objectively evaluate the effectiveness of NMES combined with swallowing-related muscle training (SRMT) curing swallowing dysfunction after stroke, so as to provide reliable evidence-based medical evidence for the clinical treatment of swallowing dysfunction after stroke.

## 2 Methods

### 2.1 Protocol registration

The protocol of this systematic review and meta-analysis has been registered on the International Platform of Registered Systematic Review and Meta-analysis Protocols (INPLASY), which has been drafted under the guidance of the preferred reporting items for systematic reviews and meta-analyses (PRISMA). Its registration number is INPLASY202110009.

### 2.2 Ethics

Not applicable. This is a protocol with no patients’ recruitment, so an approval of the ethics committee is not required.

### 2.3 Inclusion criteria and exclusion criteria

#### 2.3.1 Types of studies

Randomized controlled trails (RCTs) will be included in this study, which are related to effects of NMES for dysphagia in patients with stroke. Other studies such as case reports, reviews, retrospective studies will be excluded. And the literature retrieval will be performed in Chinese and English databases.

#### 2.3.2 Types of participants

Patients who meet the following criteria will be included in this study: adults (age over 18 years old) suffering from dysphagia after ischemic or hemorrhagic stroke. Having stroke lesion diagnosed by computed tomography (CT) or magnetic resonance imaging (MRI). The patients must be excluded a history of other neurological disorders and oral and maxillofacial surgery involving the lips and/or tongue. Patients with no limitation of type, phase, sex, nationality, race, etc.

#### 2.3.3 Exclusion criteria

The intervention for patients is NMES associated with other therapies. Poor general conditions such as severe heart, lung, and infectious diseases. That trials reporting on patients with a history of swallowing disorder before stroke diagnosis. The studies pertain Non-Chinese and English literature. Duplicate publication. Data cannot be extracted from original literature. The types of article contain review, meta-analysis, conferences, and doctoral dissertations. Unable to access full-text.

### 2.4 Types of interventions

SRMT will be utilized both in experiment group and the control group. SRMT includes tongue to jaw resistance training, tongue training, and other swallowing related muscles (for instance, submental muscle, infrahyoid muscle, and respiratory muscles) training.

### 2.5 Outcome measures

Esophagus under x-ray fluoroscopy, which is treated as the gold standard of swallowing function and total effective rate, will be evaluated as the primary outcome. Secondary outcomes contain: the Eating Assessment Tool-10 item scores, Kubota Drinking Water Test, Swallowing Capacity Test, Functional Oral Intake Scale, Videofluoroscopic Dysphagia Scale, Dysphagia Outcome and Severity Scale, and so on.

### 2.6 Search methods

#### 2.6.1 Electronic searches

Two researchers (BD and YL) will search literature independently. Including 4 Chinese electronic databases and 5 English electronic databases will be searched from the establishment of database to December 12, 2020. The Chinese literatures obtain from CBM, CNKI, WanFang, and VIP. The English literatures mainly search from PubMed, Embase, Cochrane Library, Web of Science, and ClinicalTrials. All published RCTs associated with the effects of NMES for dysphagia in patients with stroke will be selected. Exemplary search strategy of PubMed is listed in Table 1. The variety expressions of terms exist in Chinese and English retrieval, so the terms for searching will be adjusted accordingly.

**Table 1:** Search strategy in PubMed database.

Search level	Search terms
Diseases terms	
#1	((((“Stroke”[MeSH]) OR ((((((((((((((((((((((((((((Cerebrovascular Accident [Title/Abstract]) OR (Cerebrovascular Accidents[Title/Abstract])) OR (strokes[Title/Abstract])) OR (CVA[Title/Abstract])) OR (CVAs [Title/Abstract])) OR (Cerebrovascular Apoplexy[Title/Abstract])) OR (Apoplexy, Cerebrovascular[Title/Abstract])) OR (Vascular Accident, Brain[Title/Abstract])) OR (Brain Vascular Accident [Title/Abstract])) OR (Brain Vascular Accidents[Title/Abstract])) OR (Vascular Accidents, Brain[Title/Abstract])) OR (Cerebrovascular Stroke [Title/Abstract])) OR (Cerebrovascular Strokes [Title/Abstract])) OR (Stroke, Cerebrovascular [Title/Abstract])) OR (Strokes, Cerebrovascular [Title/Abstract])) OR (Apoplexy [Title/Abstract])) OR (Cerebral Stroke [Title/Abstract])) OR (Cerebral Strokes [Title/Abstract])) OR (Stroke, Cerebral [Title/Abstract])) OR (Strokes, Cerebral [Title/Abstract])) OR (Stroke, Acute [Title/Abstract])) OR (Acute Stroke [Title/Abstract])) OR (Acute Strokes [Title/Abstract])) OR (Strokes, Acute [Title/Abstract])) OR (Cerebrovascular Accident, Acute [Title/Abstract])) OR (Acute Cerebrovascular Accident[Title/Abstract])) OR (Acute Cerebrovascular Accidents [Title/Abstract])) OR (Cerebrovascular Accidents, Acute [Title/Abstract])))
#2	((((Dysphagia [MeSH Terms]) OR (Deglutition Disorder [Title/Abstract])) OR (Swallowing Disorder [Title/Abstract])) OR (Oropharyngeal Dysphagia [Title/Abstract])) OR (swallowing difficulty [Title/Abstract])
Intervention terms	
#3	((neuromuscular electrical stimulation [Title/Abstract]) OR (NMES[Title/Abstract])) OR (swallowing training [Title/Abstract])
#4	#1 AND #2 AND #3

#### 2.6.2 Searching other resources

In addition, a secondary search will be conducted to make up the deficiencies of the electronic databases. The references of included literature and relevant reviews will be scrutinized again. Through this method, a comprehensive search will be performed.

### 2.7 Data collection and analysis

#### 2.7.1 Selection of studies

**Figure 1:**
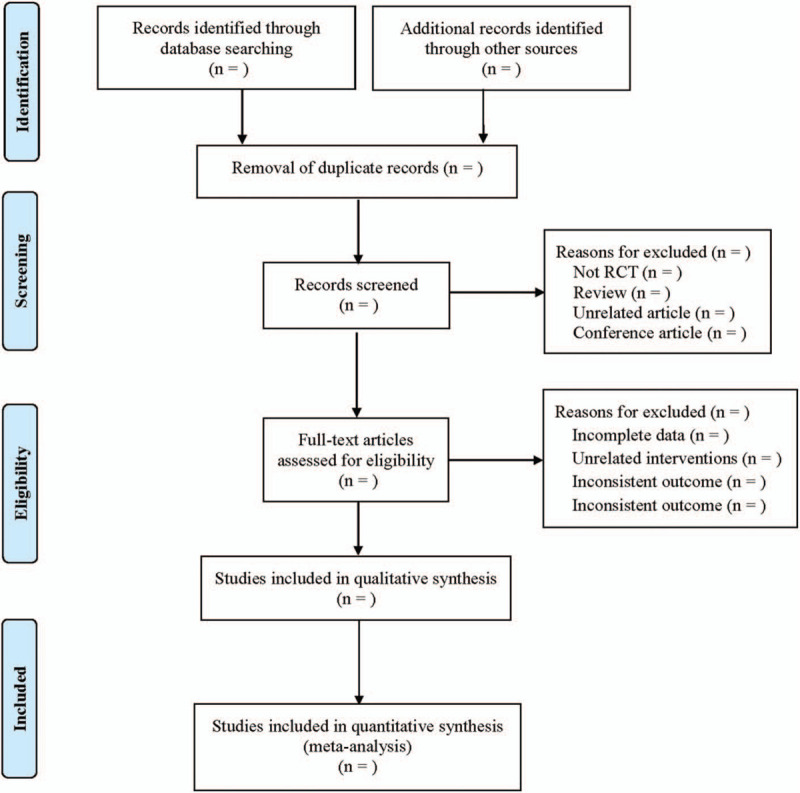
Flow chart of studies screening.

The EndNote document management software will be used to extract data. At the same time, the data will be synthesized and stored in Excel chart. Two researchers (BD and YL) will carefully read abstracts and titles of search results to identify initially included studies. Then, the full-text screening and data extraction will be conducted independently. If there are disagreements, they will be resolved by discussing or consulting another author. The process of research screening will be displayed in following flow diagram (Fig. 1), which will be performed according to the PRISMA guidelines.

#### 2.7.2 Data extraction and management

The EndNote document management software and Microsoft Excel will be used to extract and manage data. Meanwhile, the data will be synthesized and stored in Excel. These data will be extracted from researches: author, year of publication, interventions of experimental groups and control groups, time point, outcome measures, age of patients, total number of patients in the study, patients’ basic information, etc. Two researchers (BD and YL) will independently extract these data. The disagreements related to data extraction will be resolved by reaching a consensus or consulting another author LZ.

#### 2.7.3 Assessment of risk of bias in included studies

The risk of bias of the included studies will be evaluated by 2 reviewers (BZ and WZ) via using Cochrane Collaboration's tool. Considering the bias would influence our study result, so these items will be evaluated: random sequence generation (selection bias), allocation concealment (selection bias), blinding of participants and personnel (performance bias), blinding of outcome data (detection bias), incomplete outcome data (attrition bias), selective reporting (reporting bias), and other biases. The assessment for the risk of bias categorize into 3 levels: low, high, or unclear risk of bias. If there is a disagreement in judging the risk of above biases, a consultation will be taken with the researcher LZ. When >10 articles are included, funnel plot also could be used to visualize the bias of the included articles. Scattered at the bottom of the funnel plot are the studies with small sample size and low research accuracy and vice versa.

#### 2.7.4 Measures of treatment effect

In this study, statistical analysis will be conducted by using RevMan 5.3 software (developed by the UK's International Cochrane Collaboration). Risk ratio (RR) with 95% confidence intervals (CIs) will be adopted for intervention effect of dichotomous data. Mean difference (MD) with 95% CIs will be for intervention effect of continuous data. When measurement methods or units are inconsistent, the standardized mean difference (SMD) with 95% CIs will be used to present the intervention effect.

#### 2.7.5 Dealing with missing data

We will attempt to contact authors for that data that cannot be extracted. If accurate data are not available, this literature will be excluded.

#### 2.7.6 Assessment of heterogeneity

Heterogeneity of included researches will be statistically assessed by using Chi-square test (*X*^2^) and I-squared (*I*^2^) will be used to describe inconsistency quantification. When conducting data consolidation for further analysis, *P* value in (*X*^2^) test >.1 and *I*^2^ ≤ 50% will be considered there is no statistical heterogeneity so we could use the random effect model for data consolidation. Conversely, the fixed effect model will be used when there exists heterogeneity for merging the results of included studies.

If there is a significant statistical heterogeneity in data consolidation, descriptive analysis method will be utilized for analyzing those data. Potential clinical heterogeneity will be assessed by prespecified subgroup analyses. The *Z*-test is used for the overall effect.

#### 2.7.7 Subgroup analysis

If the included studies have significant statistical heterogeneity, then the subgroup analysis will be conducted basing on varied parameters that affect the result parameters. These parameters contain the characteristics of patients (for instance, the severity degree of the disease, different stage of stroke), the characteristics of interventions (for instance, total intervention duration, intervention frequency), and so on.

#### 2.7.8 Sensibility analysis

To evaluate the reliability of our study results, sensitivity analysis will be used. If there is no significant change in the results after deleting the literature, it indicates that the sensitivity is low and our results are reliable. On the contrary, if there is a big difference or even an opposite conclusion after deleting the literature, it indicates a high sensitivity and a low reliability of this study results. When interpreting the results and drawing conclusions, high sensitivity results indicate that there exist potential bias factors related to the effect of the intervention measures.

#### 2.7.9 Grading the quality of evidence

The quality of researches will be evaluated by 2 researchers (BZ and WZ) independently according to Grading of Recommendations Assessment, Development and Evaluation (GRADE) criteria. Then research data will rate into the following levels: high quality, medium quality, low, and very low quality.

## 3 Discussion

Stroke is one of the most common neurological causes of dysphagia.^[16]^ The non-drug non-surgical rehabilitation therapies that can improve swallow function includes swallowing related muscles training (for instance, lip and tongue training,^[17]^ respiratory muscle strength exercises),^[18]^ noninvasive treatments of swallowing sensation or nerve stimulation (for instance, electrical, magnetic,^[19]^ cold or sour taste stimulation),^[20,21]^ and alternative therapies (for instance, electroacupuncture,^[22]^ or acupuncture).^[23]^

Non-invasive NMES mainly stimulates local muscle contraction and/or activates muscle-related sensory pathways. When electrical stimulation performs on the peripheral nerve of the neuromuscular system, surface electrodes that could trigger muscle contraction depolarize the oropharyngeal musculature causing muscle contraction without the involvement of the central nervous system.^[16]^ The sensory axons depolarize by electrical stimulations, so a large number of signals are sent to the central nervous system. Repeated that signal stimulations can promote the central nervous system to control muscles through this pathway of the spinal cord.^[24]^ When training the lip, tongue, and muscles that involved in swallowing, the sensorimotor stimulation of these muscles controlling the nerve can be increased. Repeated those stimulations can help to create the stimulation of the central nervous system related to swallowing.^[18]^ We speculate that NMES combined with SRMT may stimulate the nerves in the swallow-related areas in the peripheral nerves and cortex, so as to use the plasticity of the central nervous system to improve the reflex of the nerves in the injured area, ultimately treating the swallowing disorders in stroke patients.

The people doing the NMES must have professional theoretical knowledge,^[25]^ and at the same time, the therapy causes a certain discomfort to the patient's pharynx,^[26]^ so its clinical application is limited. On the other hand, there are big differences in treatment effect evaluation methods, so it is difficult to accurately evaluate the clinical effectiveness of NEMS combined with SRMT therapy. This study aims to supplement the evidence-based medicine evidence of NMES therapy combined with SRMT in the treatment of patients with swallowing dysfunction after stroke by incorporating with present randomized clinical studies. However, this study still has certain limitations. Among them, the intervention time, frequency, and amplitude of NEMS are quite different, which may cause obvious heterogeneity. In addition, the search scope of this review only involves Chinese and English databases, and other language-related studies may be ignored.

## Author contributions

**Data curation:** Bosong Du, Yan Li.

**Funding acquisition:** Li Zhou.

**Methodology:** Bosong Du and Yan Li.

**Project administration:** Li Zhou.

**Resources:** Bingran Zhang and Wenjun Zhao.

**Software:** Wenjun Zhao, Bosong Du, Yan Li, and Li Zhou.

**Supervision:** Bingran Zhang, Wenjun Zhao.

**Writing – original draft:** Bosong Du, Yan Li.

**Writing – review & editing:** Bosong Du, Yan Li, Li Zhou.
